# Lessons learned from developing and sustaining a community-research collaborative through translational research

**DOI:** 10.1017/cts.2018.7

**Published:** 2018-08-08

**Authors:** Amy LeClair, Jean J. Lim, Carolyn Rubin

**Affiliations:** 1 Tufts Medical Center, Boston, MA, USA; 2 Tufts University School of Medicine, Boston, MA, USA

**Keywords:** Community engaged research, stakeholder engagement, health equity, Asian Americans, qualitative methods

## Abstract

**Introduction:**

The goal of this project was to document the current state of a community-academic partnership, identifying early successes and lessons learned.

**Methods:**

We employed qualitative methods, semi-structured interviews and document analysis, from 2 data sources to (1) show how the principles of community-based participatory research are enacted through the activities of Addressing Disparities in Asian Populations through Translational Research (ADAPT) and (2) elucidate the barriers and facilitators to adhering to those principles from the perspectives of the members themselves.

**Results:**

In addition to established community-based participatory research values, understanding individuals’ motivations for participation, the challenges aligning the priorities of community organizations and academic partners, and definitions of success are themes that emerged as key to the process of maintaining this partnership.

**Conclusion:**

As the emphasis on community-academic partnerships grows, there is potential for clinical and translational science awards to use community engagement to facilitate translational research beyond the traditional medical spheres of influence and to forge relationships with affected communities.

## Background

The emphasis on stakeholder engaged and community-based participatory research (CBPR) has been growing, as clinical and translational science awards (CTSA) use community engagement to facilitate translational research [1–3]. CBPR is a well-documented approach to addressing health disparities in minority communities [4, 5]. CBPR encourages the involvement of community stakeholders, including nonprofits, patients, and representatives from minority groups, in the research process. CBPR builds on equal, collaborative partnerships between community stakeholders and academics in all stages of the research process—from research development to dissemination—that aims to improve local public health [6, 7].

This approach has been found to be especially useful as a means to ensure translation of research findings to minority populations that are underrepresented in medical research [4, 5]. The benefit of engaging community stakeholders is that it allows research to adapt to the community’s cultural, organizational and knowledge context, develop interventions that motivate communities’ collective action, build trust between researchers and communities, and deploy sustainable interventions [2]. Therefore, researchers are able to leverage multiple perspectives in “problem/question identification, research design, research conduct, data analysis, interpretation, and dissemination of research results” [6, 7].

Given the differing agendas of academic researchers and community-based organizations (CBOs), however, sustaining such partnerships over the extended timeframes required to build trust for meaningful collaboration between the community and academics is a major challenge. While researchers are interested in generating new knowledge, communities are often more interested in practical matters, such as improving services and programs. Also, communities may want to look for solutions to meet their immediate needs, while, for academics, the road to an evidence-based evaluation of the outcomes of an intervention may require more time [8]. In addition, power imbalances between researchers and the community present another obstacle to forging an equal partnership, as factors such as knowledge and resources can affect how much influence each party has in shaping the research, how much they contribute to co-learning, how much they benefit from the knowledge generated by the research, and their long-term commitment to the partnership [9, 10].

Here we present an example of developing and sustaining such a partnership. Addressing Disparities in Asian Populations through Translational Research (ADAPT) is a community-research partnership funded by the Tufts Clinical Translational Sciences Institute (CTSI), with additional support from the Department of Public Health and Community Medicine at the Tufts University School of Medicine. Founded in November 2011, ADAPT brings together 6 Chinatown-serving CBOs and academic researchers from across Tufts CTSI and Tufts University (TU) with the goal of improving health for the local Chinatown community in Boston and beyond [11].

The goal of this paper is to present ADAPT as a model of long-term community-academic collaboration in translational research, describe the activities of the coalition, and demonstrate the benefits and challenges to CBPR from the perspectives of both community and academic members.

## Methods

We employed qualitative methods to (1) evaluate whether and how the principles of CBPR are enacted through the activities of ADAPT and (2) elucidate the barriers and facilitators to adhering to those principles from the perspectives of the members themselves.

Two major sources of data were used. Primary data collection for this study was semi-structured interviews with current ADAPT members. A purposive sample of 4 community partners and 4 academic researchers (n=8) was selected to capture a variety of perspectives, representing two-thirds of the community partners and three-fourths of the academic departments on the Steering Committee (discussed below). Community partners were selected to ensure a range of organizations was represented, who had worked with a range of academic partners on different studies, and respondents were from varying positions (e.g., not all executive directors). Academic researchers were selected from across academic departments, at varying stages in their individual careers (e.g., assistant professor vs. department chair), and based on their length of time having been involved with ADAPT.

The interview guide was developed by 2 of the authors (A.L. and C.R.) with input from other members of ADAPT in an effort to reduce bias from any one individual’s perspective or from unequal input from either the academic or community perspective. The interviewer was a newer academic member of ADAPT with a background in CBO partnerships and training in qualitative interviewing. Topics covered in the interviews included the ways in which individuals and the organizations or departments they represented had benefited from their involvement; challenges to participation; experiences with other community-academic partnerships; and hopes and recommendations for the future. Interviews were recorded and transcribed verbatim by a professional transcription service. In one case where recording was not possible, the interviewer took detailed notes. Once transcripts had been reviewed for accuracy, the audio files were deleted.

The second source of data was administrative documents, including all meeting minutes, conference summaries, bylaws, and mission statements, dating back to ADAPT’s conception in 2011. The administrative documents served to create a structure of the activities that have transpired since ADAPT’s inception. Whenever possible, we relied on documentation rather than personal experience or participation, although gaps in documentation were supplemented by the knowledge of one of the founding members, who is a primary author (C.R.). The qualitative interviews provided a more detailed understanding of the events, the meanings members ascribed to them, and context for what the principles of CBPR look like in practice.

Analysis of these 2 sources of data was conducted as follows. De-identified transcripts were coded using Dedoose analytic software. After a review of the literature, we structured the codebook around established CBPR principles: community as identity, strength-based approach, collaboration, mutual benefit, co-learning, broad definition of health, dissemination [7]. The administrative documents were coded using these deductive codes identified from the CBPR literature. Emergent themes of motivation for participation, challenges aligning the priorities of community organizations and academic partners, and defining success were added after an initial review of the transcripts, and this updated codebook containing both deductive and inductive codes was used to code the interviews. All coding was done by the same member of the team who conducted the interviews (A.L.). This research was reviewed and deemed exempt by TU’s IRB.

## Results

Analysis of administrative documents showed clear evidence of the enactment of established principles of CBPR and allowed us to document the formation and structure of this collaboration. First, we present the structure and evolution of the partnership. Next, the key themes that emerged from the interviews—an organic model of development and challenges and facilitators to the partnership—are discussed to elaborate upon those known principles, such as community as a unity of identity, equitable partnerships, ecological perspectives of health, and commitment to sustainability. Finally, we discuss the challenges and facilitators to building and sustaining this partnership identified by the members themselves, including aligning the priorities of researchers and social service providers, defining success, authentic engagement and commitment, sweat equity, and belief in the potential of the partnership.

## Structure of ADAPT

**Fig. 1:**
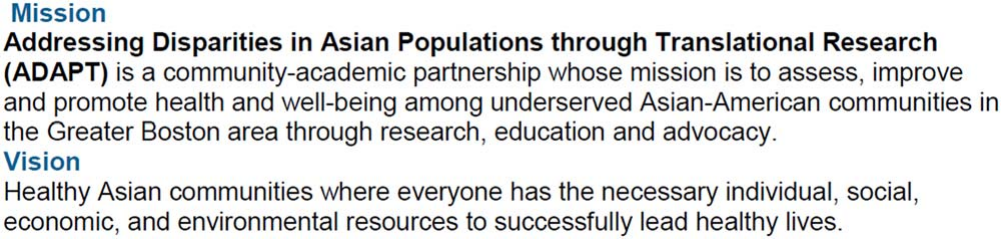
Addressing Disparities in Asian Populations through Translational Research (ADAPT) mission and vision statements.

ADAPT is organized with a director and a program manager, chosen and funded through Tufts’ CTSI, with input from the Steering Committee (see Fig. 1). There are currently 12 core members who constitute the Steering Committee: 6 from the community and 6 academic partners. Core community members “must be representatives from Asian serving community organizations that are official Tufts’ CTSI Community Partners” and academic partners must have a primary appointment at a Tufts CTSI partner institution. The 6 core community members include Action for Boston Community Development, Asian Community Development Corporation, Asian Task for Against Domestic Violence, Asian Women for Health, Boston Chinatown Neighborhood Center, and Greater Boston Chinese Golden Age Center. Each organization selects someone who is in a leadership position to represent the organization on the Steering Committee. In some cases this individual is the Executive Director, otherwise it is someone in a Program Director position. Community core members are eligible to receive stipends for attending monthly Steering Committee meetings. Over the past 6 years, ADAPT has had a consistent core of academic and community partners, and the dedication of these partners has sustained the partnership and moved it forward. ADAPT did lose 1 community partner in 2016. This particular organization does not have paid staff, so it became difficult for the co-chairs of this organization to attend ADAPT while managing competing responsibilities.

The core academic partners represent various departments and schools across Tufts Medical Center and TU, and other Tufts CTSI affiliates, including: Tufts University School of Medicine, Tufts University School of Dental Medicine, the Institute for Clinical Research and Health Policy Studies, Tisch College, the Department of Public Health and Community Medicine, the Department of Pediatrics, the Department of Psychiatry, and the Department of Medicine. New academic members that have joined over the years have frequently been referred by existing members because of their expressed interest in Asian health and/or CBPR work. There have been several academic partners who work on specific projects, but do not come to the monthly meetings where issues of our overall partnership are discussed and worked out. It is important to note that the core academic partners who do come to monthly meetings do not have salary support to do so. The number of core members is not capped, but the number of *voting* members is limited to maintain a 50/50 ratio between academic and community partner categories.

**Fig. 2:**
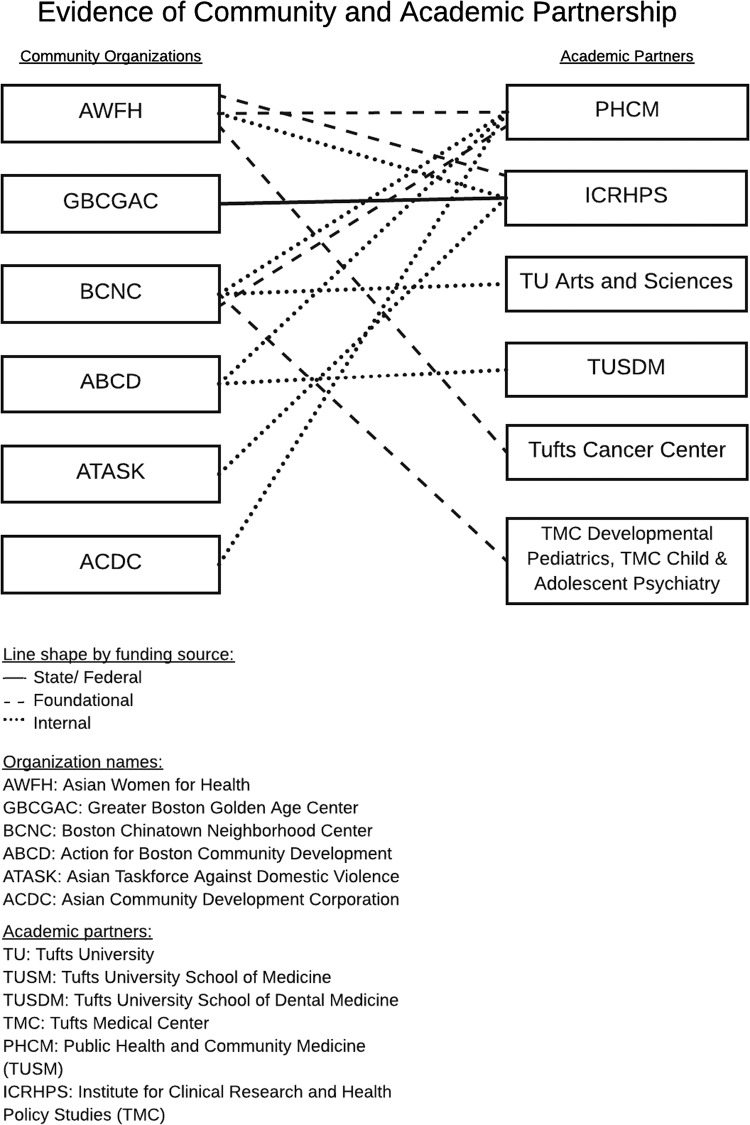
Evidence of community and academic partnership.

Over time, ADAPT has embraced and promoted a broad definition of community health. Five of the six core community partners in ADAPT do not focus explicitly on health. They are social service organizations, focused on affordable housing, job training and employment, legal services, elder services, education, childcare, family programming, and more. On the academic side, ADAPT’s members have degrees in medicine, dentistry, public health, and education, just to name a few. Although none of the community partners have “health” in their mission statement, because they understand the impact of the social determinants of health on the local community—including housing, employment, education, nutrition, among other factors—the research collaborative was able to leverage the knowledge and expertise of the academic researchers and the community partners to focus on health topics most salient to the local Chinatown community. Since its inception, ADAPT has supported over 20 successful grant applications to foundations, internal funding sources, and federal funding sources with letters of support and other resources (see Fig. 2).

## Evolution of the Partnership

The establishment of ADAPT came at an opportune moment. As written about in the partnership’s first manuscript [11], academic and community partners came together in November 2011 to explore the potential of a partnership. In December 2011, the external advisory board of the Tufts CTSI recommended to the Community Engagement Core that it focus on Tufts’ host community. The conditions were favorable for establishing the partnership: the political will of academic and community partners was present, and senior leadership of the CTSI supported its development and continuation. Since ADAPT’s formation, the Tufts CTSI has provided the majority of the infrastructure support for this partnership, which includes funding for the Director, a Project Manager, and a yearly Symposium. Community partners are provided modest stipends. The Tufts University School of Medicine, Department of Public Health and Community Medicine funds the Asian Health Equity fellow, a fellowship for a graduate student interested in learning about health equity work. The different projects that have been incubated through ADAPT have gotten their own research money, but that money funds the projects; it does not go to ADAPT.

When ADAPT was founded, it provided a regular meeting space for academic and community partners to come together and discuss issues facing the Chinatown community. This first phase (from 2011 to 2015) focused on building trust between Tufts and the Chinatown community and developing a shared language around Chinatown health priorities. In this first stage, and as a way for Tufts to demonstrate to Chinatown its commitment to community engagement, Tufts held a day-long capacity-building training based on the program, “Building Your Capacity” [11, 12].

During this initial phase, ADAPT also functioned as an “incubator” for research partnerships that were either researcher-initiated or community-initiated. Researcher-initiated projects focused on cancer and diabetes, given that these are 2 of the most documented health disparities facing the Asian community. Community-initiated projects focused on topics with less prior research, including domestic violence and nutrition and physical activity. ADAPT also supported the first population-based community survey of health needs and priorities in the Chinatown neighborhood. The first phase was also marked by yearly stakeholder forums with themes such as, “Together: Strengthening the Health of Chinatown” and “From Collecting Data to Collective Impact.” These forums grew from an audience of 35 people in 2011 to over 90 people by 2014.

During these first 4 years, the academic partners demonstrated that they were committed to improving the health of Chinatown and the broader Asian American community. Academic partners provided educational workshops, supported community events and took on community-identified research projects without salary support. Tufts students also worked on projects for which there was no funding.

In the fall of 2015, ADAPT began a strategic planning process and entered its second phase. During the strategic planning, ADAPT added a second tier of “affiliated” members who do not have voting power and are not held to the same attendance and participation standards as the core members. This second level of membership allows ADAPT to expand its engagement with diverse stakeholders and bring in new members while honoring the time and commitment made by those who have been involved for a long time. Monthly Steering Committee meetings have continued since ADAPT’s inception. The meetings are open to anyone interested, and the attendance includes both members of the Steering Committee, other (affiliate) members, trainees, and interested faculty and community.

**Fig. 3:**
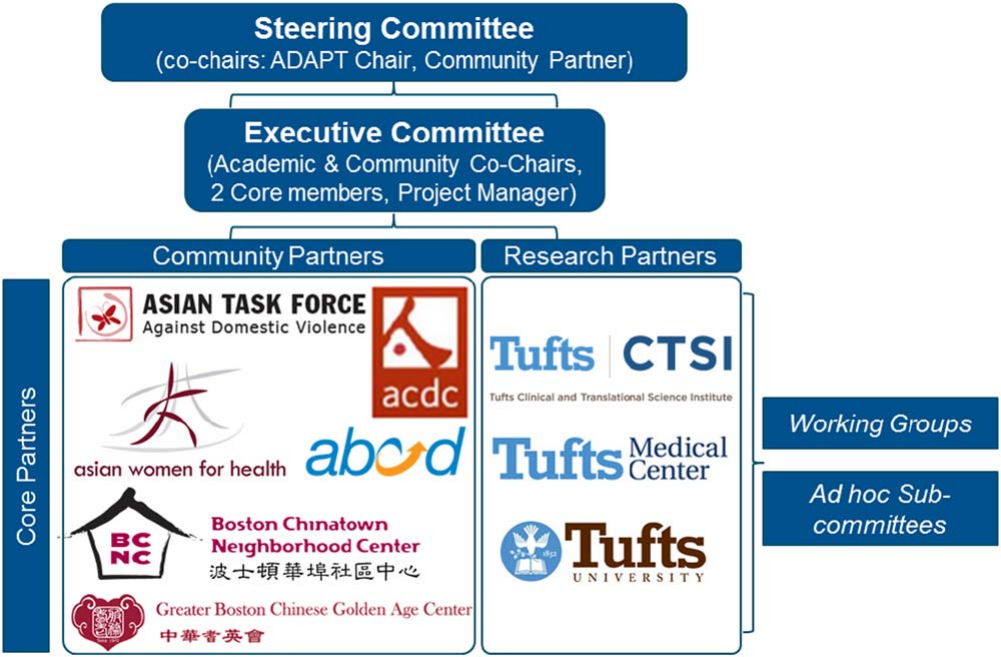
Addressing Disparities in Asian Populations through Translational Research (ADAPT’s) governance structure.

As the collaborative coalesced and trust was established, some community partners indicated that they wanted to think about a community-identified health agenda for Chinatown. They wanted to move away from a model of just being responsive to funding opportunities and ensure response to funding included the community agenda. During the strategic planning process, ADAPT refined its vision and mission statements and established a governance structure and bylaws (see Fig. 3). This included the formal creation of an Executive Committee and Steering Committee, which meet monthly.

ADAPT also identified strategic goals and priorities and developed working groups to move this work forward. There are currently 4 working groups: Diabetes Prevention, Child Health & Wellness, Collective Impact (data equity), and Stable Housing & Health. Working groups help to support research in progress, provide feedback on manuscripts in preparation, and support new funding applications. They also provide an opportunity for more in-depth conversations that are not always possible in the larger Steering Committee meetings.

## Organic Model of Partnership Development

Up until the strategic planning process in the fall of 2015 when ADAPT established a governance structure and an Executive Committee and Steering Committee, ADAPT had grown organically. The growth was fueled by the relationships that were built between academic and community partners. In the first phase, the way ADAPT approached the work was largely through smaller pilot projects. These projects accomplished several things: forged relationships between individual investigators and organizations, provided data on subsets of the community, and established a track record of collaboration among members.

At the same time, there were some moments of frustration about the lack of clear focus and direction. Community partners had voiced that they did not want so many meetings focused on grants. While the prior absence of a formal structure had allowed ADAPT to be flexible, it had created challenges as well. For example, when the group decided to pursue a major funding opportunity, there existed no formal decision-making process or voting structure. The partners were able to make decisions on good faith and trust, but as the group matured, a more formal structure was necessary.

The strategic planning process also served as another activity that reinforced the group’s intention, and members of ADAPT found the focus provided orientation and increased the commitment to the collaborative to move forward on this mission:

*Having gone through a formal process of the mission and vision… I don’t know that our work is going to always roll up exactly under what all these structures that put into place, but by virtue of doing them that we have declared that we’re serious*.

Community partner

Even if the formal structures ADAPT designed and adopted need to be revised in the future or do not fully encapsulate the work of the partnership, engaging in that process served to solidify some of ADAPT’s structure.

Relationships built in the first phase helped to reinforce members’ commitment to ADAPT and reified the partnership’s purpose. Reflecting on the strategic planning process, one academic member said:

*I think that’s part of the evolution of the group, and if we had forced ourselves to work together on something to establish a track record from the beginning, that would have been probably a mistake, because we would not have built the dynamic of the group…we might have ended up in a place that was more top-down.*

While the lack of formal structure sometimes created challenges, this member articulated how it allowed for ADAPT to form organically. If the group had forced itself to formalize before it was ready to do so, the outcome may have felt very different.

## Challenges and Facilitators to the Partnership

As mentioned, while ADAPT has enjoyed modest success, it is not without its challenges. These challenges include aligning the needs of different members and defining success within the partnership. Finally, we present some of the qualities of ADAPT that have enabled its success, including an authentic engagement in this type of work, “sweat equity,” and the shared belief in collective potential.

### Aligning the Priorities of Researchers and Social Service Providers

Like other CBPR partnerships, ADAPT has struggled with the tension between research and service. While ADAPT academic and community partners are united around a vision of addressing Asian health disparities and achieving health equity, how they approach the work differs. The role of academics is to produce new, generalizable knowledge. In the words of one academic member, “academics don’t really advocate in the same way that the agencies advocate.” Another way in which this mismatch manifests itself is in regards to time. Service agencies want research to have an immediate impact on their clients. For example, they are often not satisfied with formative or exploratory research. They often want research that can rapidly lead to new interventions or evidence-based practice—this was made clear in both discussions captured in minutes from Steering Committee meetings and annual research symposia. The pace of academic research—applying for grants, receiving funding (or not), collecting and analyzing data, publishing and disseminating results—may not align with the timeline on which community organizations operate. Striking a balance between these potentially disparate sets of needs and institutional demands has been a consistent tension throughout ADAPT’s history.

One of ADAPT’s strengths is the diversity of its membership. The diversity of research interests and expertise of the researchers, however, does not necessarily align with the health topics identified as priorities to the community:

*There’s the intrinsic problem that you can go through the process of saying these are the things that are important to the community…but that doesn’t mean we have the infrastructure to actually work on those, so that’s a limitation that really could only be addressed by having enough finances to bring in different kinds of researchers*.

Academic partner

As a concrete example in ADAPT, the community partners have identified mental health as an area of concern for their client populations. The current lack of established investigators within this content area among the academic partners has been a limitation to move this agenda forward. The collaborative is exploring ways to bring in external expertise and/or develop it among current partners to address this need. Having a broad definition of health that focuses on not just the absence of disease but the presence of wellness and well-being does not always align with dominant funding mechanisms, which are often disease-focused. In the words of one academic member, “Some of the parameters for research are too constrained or too narrow.” Documents from Steering Committee meetings, including invited presentations and group discussions, and from working groups, however, demonstrate that the group is finding ways to incorporate mental health and wellness into its work.

### Defining Success

Because the overall mission is broad in terms of improving health and wellness in the community, and because much of the early work of ADAPT involved the process of defining and developing the partnership, it is not clear what the definition of “success” is, or what the specific benchmarks and outcomes are, though members are committed to being at the table—literally and figuratively—for now. The same members who discuss the dangers of not having a tangible product, in terms of grants or publications, also recognize the limits of focusing too much on outcomes:

*Maybe that’s important, not to over expand your thinking of what [success is]. We’re a committed group that definitely wants to do projects that represent large enough segment that it makes sense for us to work together, and we really could do something together that would make a difference.*

Academic partner

In addition to assessing success in terms of the process of the group versus the outcomes, the 2 partner groups have differing foci on success. Community members define success in terms of programs and services offered to address community needs. Though the academics are committed to ADAPT, they are under institutional pressure to bring in grants and produce publications. One academic member said:

*I think that’s a real danger that without some success, some grant funding coming through, if that truly is sort of how we’re being measured in terms of success, because I feel like on the academic side, that’s still how we’re getting measured.*

Thus far the academic and community members have been able to balance those competing definitions, in part through the smaller grants mentioned earlier.

### Authentic Engagement and Commitment

Several members—both community partners and academics—noted that their motivation for continued participation was based in part on their affinity for the other members. If people were not having fun, they would not keep coming. One academic member noted seeing this concept of “affinity” in the CBPR literature:

*I used to always think, “Somebody needs to research this.” I think affection develops. And I think we’ve got it. I’ve seen it now in some of the coalition literature. The ones [coalitions] that work, there’s affection. And I think we’ve got it at ADAPT…it’s a kind of a combination of available interests and skills with the opportunities, and you got to stick together until that all converges. And we got to hang in there until it converges, and I think it will converge again. But I think it’s kind of the affection that keeps you together.*

Indeed, many of the academic partners had experience working with community organizations before joining ADAPT. One researcher joined because of a self-proclaimed natural affinity for this type of work: “one of my big attractions to ADAPT was that when I came here [Tufts], I left my community partners behind. And it was really my favorite part of the work.” The members find this work personally rewarding, even if they have not “benefitted” in ways that build their promotion portfolio: “I don’t know if I have benefitted. I mean I benefitted because I think it’s important, it keeps your research more real.” They believe in this type of work, and that it does make them better researchers:

*I think that it has helped me be a better researcher because I do think that community/academic partnership really does result in asking better research questions and moving the research agenda forward.*

Academic partner

Enjoying the work, however, cannot sustain an academic salary or fund an organization: “If these grants don’t come through or whatever and my time isn’t supported, it’s just really hard to continue to justify it [participation] at the departmental level.” There is not a clear expiration date on this partner’s involvement, but addressing this challenge of unfunded time will continue to be part of the work of this group as a collective and as individual members.

At the same time, the community partners recognize that academics are committed and care about the community. When asked what they think works well about ADAPT, one community partner said:

*Because it’s a regular group that comes together, and the researchers that come are not necessarily researchers that have an active project, but just people who, when they want to be able to do an active research project in that community, want to be able to go in with some knowledge.*

Community partner

Sensing an authentic commitment to the community, another community partner talked about other groups their organization had been involved with and noted, “it’s very clear that the agenda is set by the large body, the one that is the convener.” This community partner felt it was different with ADAPT, where “there is a lot of influence that any individual community member can have on the agenda and the direction of the coalition by being engaged.”

### Sweat Equity

For ADAPT, commitment to Asian health equity is demonstrated by being physically present and involved. Funded or not, there is no substitute for showing up and doing the work, and the academic partners know this.

*I just feel like you really have to put literally, the time in in order to get your street cred. And you can apply theory and check all the boxes, and that’s important, too, but at the end of the day it’s really developing those relationships, and then following through, which takes time, and you’re not getting paid for it.*

Academic partner

Academic partners are aware of the pitfalls of CBPR cited in the literature and strive to avoid those. They understand opportunistic engagement does not work: “This stuff is built on trust which doesn’t come overnight.” Knowing this is an important motivator for academic partners. They recognize the importance of trust and that it must be earned. In describing how they benefit from participating, one academic partner talked about the importance of showing up: “You’re at ADAPT, I can trust you. I know you’re serious about this work. So I think it connotes some amount of seriousness and goodwill.”

This active investment and engagement makes a difference in building trust. Because of the investment that academic and community partners have put into ADAPT over the past 6 years, a tension has arisen on how to reach out and integrate new members and potentially expand the reach of ADAPT while acknowledging and honoring the commitment of those academic and community partners that have been at the table from the beginning. These tensions are captured in the discussions held during monthly meetings.

### Belief in Potential: Leveraging Our Collective Power

As the group has evolved, however, it has become increasingly clear that ADAPT wants to work on a project or initiative that can be worked on across agencies. In the words of one community member:

*I do feel sometimes it’s a group in search of a project. It’s a chicken and egg problem…I, enjoy, I sense that people enjoy the meeting in part, but then if it doesn’t bear fruit then is becomes less enjoyable.*

As the group continues to pursue larger grants, the challenge is to do what we can with what we have. As one community member phrased it: “What can we do with no money to set ourselves up for a successful future?”

The fact that members continue to come to the table despite the lack of adequate resources is a testament to the commitment of the individual partners to the larger vision and mission. Many stay involved because they recognize that ADAPT’s greatest strength is its potential. In the words of one community partner, “There’s a shared dedication to what can potentially be done in the future with this coalition, people continue to engage.” ADAPT’s challenge is to realize that potential. Another community partner remarked, “I stay involved with ADAPT because I think we can go towards something really groundbreaking, but we’re not necessarily breaking ground right now.”

In the past year, ADAPT members voted to focus their efforts on a “collective impact” project, an initiative designed to collect some standardized outcome measures across agencies in Chinatown in order to assess their organizations’ impact on the overall health of the neighborhood and to identify service gaps. Evidence of this effort is seen in both the creation of the “Collective impact” working group and the Steering Committee’s voting to make collective impact the focus of the partnership’s work this year. Though there is currently no precise funding opportunity soliciting applications for this work, ADAPT members have been adamant that they want to work on this issue. Embracing this as part of the core strategy in moving forward reflects a desire to enable the community partners to drive the agenda for ADAPT.

## Discussion

As the emphasis on community-academic partnerships grows and CTSAs use community engagement to facilitate translational research [1–3, 13], organizations like ADAPT are receiving more attention [14]. There is potential for CTSAs to use community engagement to facilitate translational research beyond the traditional medical spheres of influence and to forge relationships with affected communities, building their capacity to realize the potential of research to impact health.

In this paper, we describe the development and ongoing progress of a community-academic partnership with Tufts and the local Chinatown community. After 6 years, the partnership has an established organizational structure that acknowledges the strengths both of the academic members and community partners. The activities of ADAPT embody the principles of CBPR; the perspectives of the members themselves elucidate the benefits and challenges to putting these principles into practice. In addition to viewing community as identity, taking a strength-based approach, collaborating, striving for mutual benefit, co-learning, adopting a broad definition of health, and disseminating the work, understanding individuals’ motivations for participation, the challenges aligning the priorities of community organizations and academic partners, and definitions of success are themes that emerged as key to the process of maintaining this partnership. Interviews with a sample of members of ADAPT revealed themes about the working of the coalition. CBPR principles, including mutual respect, transparency, and commitment, are viewed as necessary to initiate new research collaborations, but not sufficient. While ADAPT has proven itself as a viable partnership that can respond to funding opportunities, it has become more than that. It has also become a community in unto itself. Trust and commitment—both with other members and with the group as a work in progress—are highlighted as being a necessary characteristic of participants. Time and funding are two of the most important resources, and the majority of members agree that there is no substitute for “skin in the game.” Attempts at last minute, opportunistic engagement were provided as examples of what had not worked. One ongoing tension is the balance between process and product. Individual members are beholden to organizations or departments to different degrees, and feel the need to produce something in the form of publications or grant money in order to justify the continued commitment to the collaboration. At the same time, these products are unlikely to materialize if members are not invested in the process of growing and sustaining the coalition.

There are limitations to this study. As the interviews and coding were conducted by a single researcher, who is a member of ADAPT, there may have been some social desirability bias in the respondents’ answers. The findings may not be generalizable to other groups.

The goal of this project was to document the current state of ADAPT, demonstrating how CBPR principles are enacted and elaborating on the barriers and facilitators to that work in this context. By sharing ADAPT’s story and speaking honestly and openly about our struggles, we hope to aid other collaborations who are currently engaged in or planning to embark on similar work with underserved communities, while also contributing to the broader literature on the role of coalitions engaged in CBPR.

## References

[1] Leshner AI, et al Committee to Review the Clinical and Translational Science Awards Program at the National Center for Advancing Translational Sciences. Board on Health Sciences Policy. Institute of Medicine. The CTSA Program at NIH: Opportunities for Advancing Clinical and Translational Research: Washington, DC: The National Academies Press, 2013. 24199260

[2] Wallerstein N, Duran B. Community-based participatory research contributions to intervention research: the intersection of science and practice to improve health equity. American Journal of Public Health 2010; 100(Suppl. 1): S40–S46. 20147663 10.2105/AJPH.2009.184036 PMC2837458

[3] Burke JG, et al PCOR, CER, and CBPR: alphabet soup or complementary fields of health research? Clinical and Translational Science 2013; 6: 493–496. 24330697 10.1111/cts.12064 PMC5350967

[4] Sankaré IC, et al Strategies to build trust and recruit African American and Latino community residents for health research: a cohort study. Clinical and Translational Science 2015; 8: 412–420. 26094679 10.1111/cts.12273 PMC4626334

[5] Mulvaney-Day NE, et al Developing systems interventions in a school setting: an application of community-based participatory research for mental health. Ethnicity and Disease 2006; 16(Suppl. 1): 107–117. 16681134

[6] Minkler M, Wallerstein N. Community-Based Participatory Research for Health: From Process to Outcomes. San Francisco, CA: John Wiley & Sons, 2011.

[7] Israel BA, et al Review of community-based research: assessing partnership approaches to improve public health. Annual Review of Public Health 1998; 19: 173–202. 10.1146/annurev.publhealth.19.1.173 9611617

[8] Wallerstein NB, Duran B. Using community-based participatory research to address health disparities. Health Promotion Practice 2006; 7: 312–323. 16760238 10.1177/1524839906289376

[9] Israel BA, et al Critical issues in developing and following community based participatory research principles. Community-Based Participatory Research for Health 2003; 1: 53–76.

[10] Rubin CL, et al Creating a culture of empowerment in research: findings from a capacity-building training program. Progress in Community Health Partnerships: Research, Education, and Action 2016; 10: 479–488. 10.1353/cpr.2016.0054 PMC5529141 28230555

[11] Rubin CL, et al “We make the path by walking it”: building an academic community partnership with Boston Chinatown. Progress in Community Health Partnerships-Research Education and Action 2014; 8: 353–363. 10.1353/cpr.2014.0046 PMC4519822 25435562

[12] Rubin CL, et al Community-engaged pedagogy: a strengths-based approach to involving diverse stakeholders in research partnerships. Progress in Community Health Partnerships-Research Education and Action 2012; 6: 481–490. 10.1353/cpr.2012.0057 PMC4415703 23221294

[13] Zerhouni EA. US biomedical research: basic, translational, and clinical sciences. JAMA 2005; 294: 1352–1358. 16174693 10.1001/jama.294.11.1352

[14] Rosenblum D, Alving B. The role of the clinical and translational science awards program in improving the quality and efficiency of clinical research. CHEST Journal 2011; 140: 764–767. 10.1378/chest.11-0710 PMC3168860 21896519

